# Electrochemical characterization of leached steel-making sludge

**DOI:** 10.1038/s41598-022-20980-4

**Published:** 2022-10-06

**Authors:** Šárka Langová, Bruno Kostura, Pavel Raška, Dalibor Matýsek, Vlastimil Novák, Michal Ritz, Jiří Krčmář

**Affiliations:** 1 Department of Chemistry, VSB-Technical University of Ostrava, 17. listopadu 2172/15, 70800 Ostrava-Poruba, Czech Republic; 2 Department of Occupational and Process Safety, VSB-Technical University of Ostrava, 17. listopadu 2172/15, 70800 Ostrava-Poruba, Czech Republic; 3 Institute of Geological Engineering, VSB-Technical University of Ostrava, 17. listopadu 2172/15, 70800 Ostrava-Poruba, Czech Republic; 4 Department of Physical Chemistry, VSB-Technical University of Ostrava, 17. listopadu 2172/15, 70800 Ostrava-Poruba, Czech Republic; 5 Liberty Ostrava a.s., Vratimovská 689/117, Kunčice, 719 00 Ostrava, Czech Republic

**Keywords:** Chemistry, Materials science

## Abstract

In this work, the electrochemical properties of the leached sludge, magnetite and zinc ferrite were studied. Acetic acid was used as a leaching reagent because, in recent years, there has been a surge of interest in using zinc-containing materials as photocatalysts, with acetic acid finding application in their preparation. Various methodological approaches were used, but the best results were achieved with a combination of 1–3 h leaching in 0.01 M acetic acid with a solid/liquid ratio of 500. In this arrangement, zincite was almost completely removed from the sludge, while zinc ferrite and magnetite remained in the solid residue. Ex situ analyses of the main leaching products were performed by X-ray diffraction, infrared spectroscopy, and thermogravimetry. The electrochemical behaviour of solid residue and model systems, that are micromagnetite and zinc ferrite, was studied in alkaline media by means of modified carbon paste electrodes, cyclic voltammetry, and chronocoulometry, with a suitable potential window ranging from 0 to 1.5 V. In summary, a linear dependence of the anodic and cathodic peak height on the square root of the scan rate was found. The position of the anodic and cathodic peaks shifted slightly with scan rate, only at low rates, up to 25 mV/s, the individual peaks coincided. The electrochemical response suggested a quasireversible process.

## Introduction

It is widely acknowledged that steel-making wastes are classified as hazardous since they contain heavy metals. One of them is zinc, which is contained either in the form of the highly soluble zincite ZnO or the hardly soluble zinc ferrite ZnFe_2_O_4_. Zinc and its compounds are recovered from these waste materials by acid or alkaline leaching at high or low pressures. Under atmospheric pressure, zincite can be almost selectively separated by leaching in NaOH, NH_4_Cl, or (NH_4_)_2_CO_3_, with zinc ferrite remaining in the residue^1–3^. During acid leaching, zinc ferrite also dissolves, but iron enters the solution^4–6^. This was overcome by Siedlecka^7^ and Maia^8^ who used ethyl alcohol after acid leaching to precipitate iron sulphate compounds. In addition, calcium compounds are converted to insoluble gypsum, and magnetic separation yields magnetite and hematite.

Zinc can be obtained from zinc ferrite by acid leaching under elevated pressure^9^ or using pyrometallurgical processes, such as the Waelz kiln process and its modification and the RecoDust process based on the reduction of heavy metal–containing basic oxygen furnace dust by H_2_ and CO, as described in^10^ and^11^, respectively. Pickles^12^ investigated the selective reduction by iron, reporting an optimal temperature and pressure range for zinc and lead recovery. The combination of reduction, roasting, acid leaching, and magnetic separation was proposed in^13^. The roasting of zinc leaching residues by ammonium sulphate was described in^14^, where the introduced three-step process provided a highly pure residue suitable for recycling in ironmaking. Kashyap and Taylor^15^ used H_2_ gas to partially reduce zinc ferrite. It is also worth noting that zinc can be selectively separated from franklinite by a combined thermal—hydrometallurgical process using NaOH^16–18^.

Steelmaking wastes and their leaching products are of great practical importance. Steel-making sludge can be used as raw material for ceramic production^19^ or to remove heavy metals from wastewater, as shown in^20,21^. After adding lime, briquettes can be made from the sludge and used in the converter^22^. Roslan et al.^23^ investigated the possibility of improving the properties of pozzolanic cement derived from by-products of steel manufacturing industries. Zinc ferrite is widely studied as a component of a hybrid nanocomposite that exhibits superparamagnetism, and as a catalyst for various chemical reactions. The photocatalytic application of zinc-containing materials is discussed in several works^24–32^. Especially ZnO-graphene oxide nanohybrid exhibited excellent photocatalytic properties during the photodegradation of crystal violet. The electrochemical study of the zinc ferrite-based sensor was carried out in^33–36^. The supercapacitive behaviour of ferritic materials was investigated in^37^. The nanoparticles retained more than 87% of the initial capacitance after 1000 charge/discharge cycles. The zinc ferrite-based graphene nanocomposites were tested as promising electrocatalysts by Nivetha and Grace^38^. MnFe_2_O_4_/graphene and ZnFe_2_O_4_/graphene nanocomposites were found to be efficient electrocatalysts for hydrogen generation via the hydrogen reduction mechanism.

This study makes several contributions to the matter of sludge acid leaching and solid residue characterization. It extends them by investigating the behaviour of a carbon paste electrode modified with sludge leached in acetic acid to assess the stability and reversibility of this system. Acetic acid was chosen to separate zincite because zinc acetate can be used to prepare nanocomposites with photocatalytic effects, as demonstrated in^39^. The influence of the liquid/solid ratio, time, and acid concentration on the selectivity of zinc leaching has already been investigated elsewhere^40^. Magnetite and zinc ferrite were used as model modifiers. Although the processes in a system comprising an inert working electrode and a solute are relatively well-known, the behaviour of solid electrodes consisting of metal in different oxidation states is poorly understood. Electrochemical reactions of iron compounds could be of great importance for green metallurgy and other environmentally friendly technologies.

## Experimental

The dried sludge from the open-hearth furnace was used for the leaching experiments. The particle size was less than 0.1 mm. Electrochemical and thermal characterization was also performed with zinc ferrite (Alfa Aesar, 99%) and micromagnetite from Sigma-Aldrich. The samples were leached in the acetic acid solutions. The content of the monitored elements was determined using AAS (Varian AA280FS) and is shown in Table 1. All other elements were less than 1%.

**Table 1:** Chemical composition of the steel-making sludge (wt%).

Zn	Fe	Pb	Cd	Mg	Ca	Mn	Cr
9.5	54.0	0.72	0.01	0.33	0.65	0.91	0.07

The leaching experiments were carried out at ambient temperature (22 ± 2 °C) in a shaker at 180 rpm. The effect of the acid concentration, the liquid/solid ratio, and time on the selectivity and metal extraction was studied. Selectivity was defined as the ratio of zinc and iron extraction.

Mineralogical analysis was carried out using a Bruker-AXS D8 Advance instrument with a 2θ/θ measurement geometry and the positionally sensitive detector LynxEye. It was described in detail in^40^.

Thermal analysis was carried out using a TA Instruments Discovery SDT 650 simultaneous thermal analyser with autosampler at a heating rate of 5 °C min^−1^ and in an air atmosphere. The weight of the sample for analysis was 20 mg.

Electrochemical measurements were performed on a VoltaLab 40 PGZ301 potentiostat (Radiometer Analytical, France). A three-electrode electrochemical system was used with the modified carbon paste electrode (CPE), platinum wire, and SCE electrode serving as working, counter, and reference electrodes, respectively.

The graphite powder (CR 5 product with an average particle size of 5 µm, Maziva Tyn nad Vltavou, CR) and high-purity paraffin oil (Fluka) serving as a nonelectrolyte binder were used for the preparation of carbon paste electrodes. The modified paste had the following composition: 59 wt% graphite powder (particle size < 5 μm, 95%, Sigma-Aldrich), 26 wt% spectroscopic paraffin oil, and 15 wt% modifier. Micromagnetite, zinc ferrite, and steel-making sludge after acid leaching were used as modifiers. The preparation and use of the carbon paste electrode is described in^41^, where the possible reaction of iron and iron oxides in 1 M NaOH were studied.

The Raman spectra of the samples were measured on a DXR SmartRaman dispersive Raman spectrometer (Thermo Scientific, USA) with a CCD detector using a 180° measurement technique without sample preparation. The measurement parameters were as follows: excitation laser 780 nm, grating 400 lines/mm, aperture 50 μm, exposure time 1 s, number of exposures 250, and the spectral region 2000–50 cm^−1^. An empty sample compartment was used for background measurement. Treatment of spectra: fluorescence correction (6th order).

## Results and discussion

**Figure 1:**
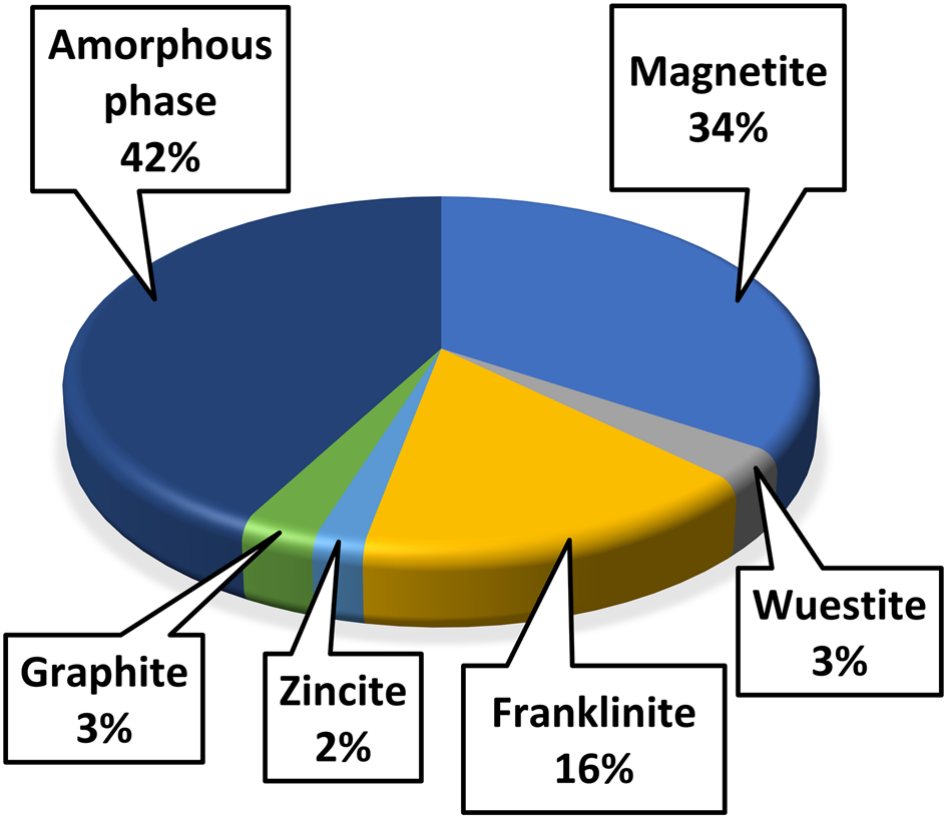
Mineralogical composition of the sludge.

Figure 1 presents the mineralogical composition of the original sludge. It must be noted that the evaluation error for magnetite and franklinite content can be quite high because of their similar structure.

**Figure 2:**
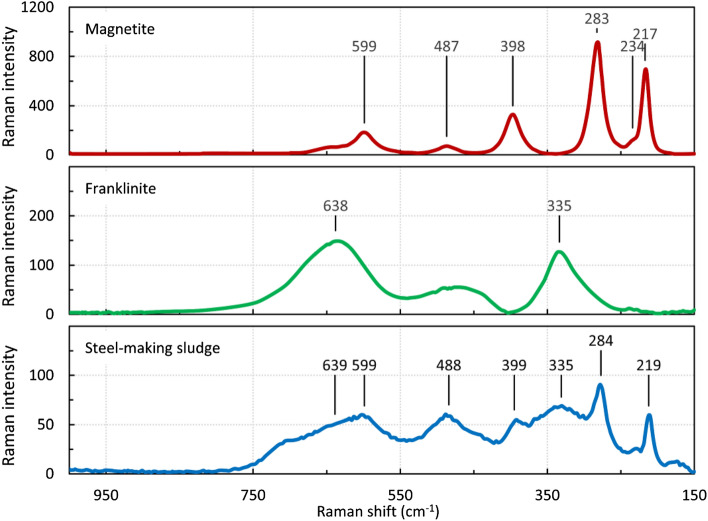
Comparison of Raman spectra of original sludge, franklinite, and magnetite.

Raman spectroscopy confirmed the presence of magnetite and franklinite as the main crystalline phases of steelmaking sludge. Figure 2 compares the Raman spectra of the steelmaking sludge with those of pure magnetite and franklinite. The spectra of steelmaking sludge contain spectral bands at 219 cm^−1^, 284 cm^−1^, 335 cm^−1^, 399 cm^−1^, 488 cm^−1^, 599 cm^−1^, and 639 cm^−1^. The spectral bands at 219 cm^−1^, 284 cm^−1^, 399 cm^−1^, 488 cm^−1^, and 599 cm^−1^ are characteristic of the iron oxide spectrum (see magnetite spectrum). The other bands in the sludge spectrum (335 cm^−1^ and 639 cm^−1^) are typical of the franklinite spectrum.

The chemical composition is listed in Table 2.

**Table 2:** Chemical composition of the solid residue (wt%).

Zn	Fe	Pb	Cd	Mg	Ca	Mn	Cr
8.0	59.0	0.63	0.01	0.01	0.19	0.92	0.08

**Figure 3:**
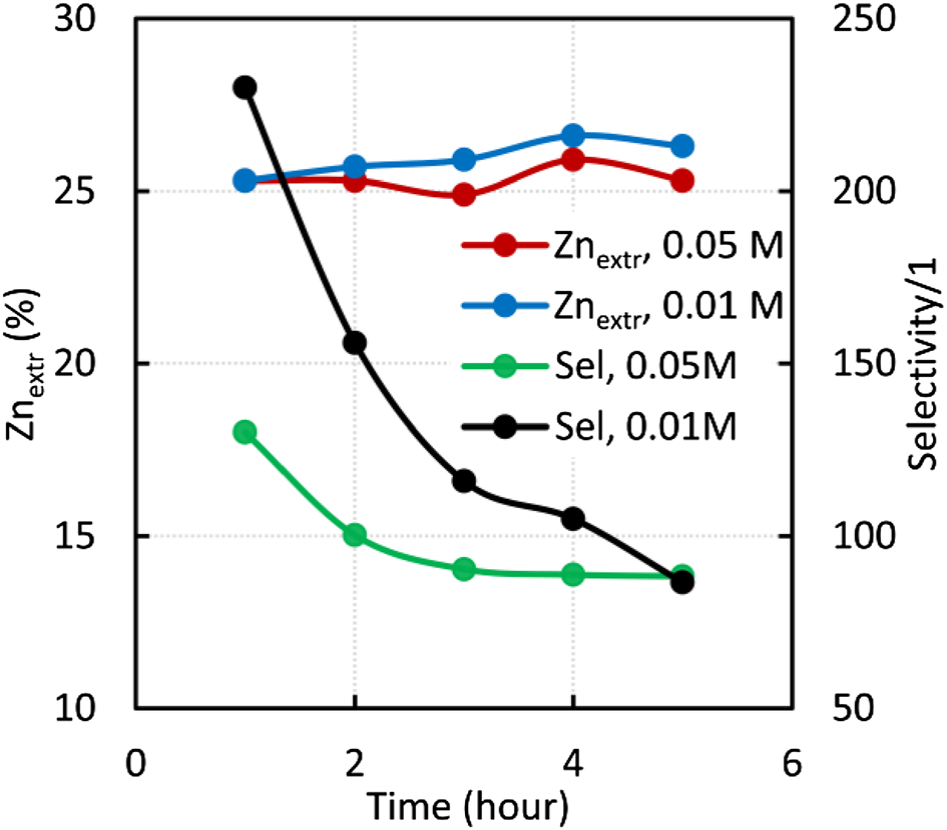
Time dependence of metal extraction and selectivity of the sludge (0.1 g) in 0.5 mol of acetic acids.

Figure 3 shows the results of investigating the effect of acid concentration and time on the selectivity and extraction of zinc^40^. Sludge weighing 0.1 g was leached in the acetic acid solutions − 10 ml of 0.05 M solution and 50 ml of 0.01 M solution. It can be seen from Fig. 3 that zinc extraction is almost independent of the acid concentration, provided that the amount of substance is sufficient to extract zincite. On the other hand, the selectivity decreases markedly with increasing acid concentration. Similar results were obtained during our previous experiments with sulphuric and hydrochloric acid. Thus, leaching in the diluted acids can be applied to distinguish the zinc fixed in zincite and franklinite. Zinc extraction did not increase during more extended experiments, but selectivity decreased.

**Figure 4:**
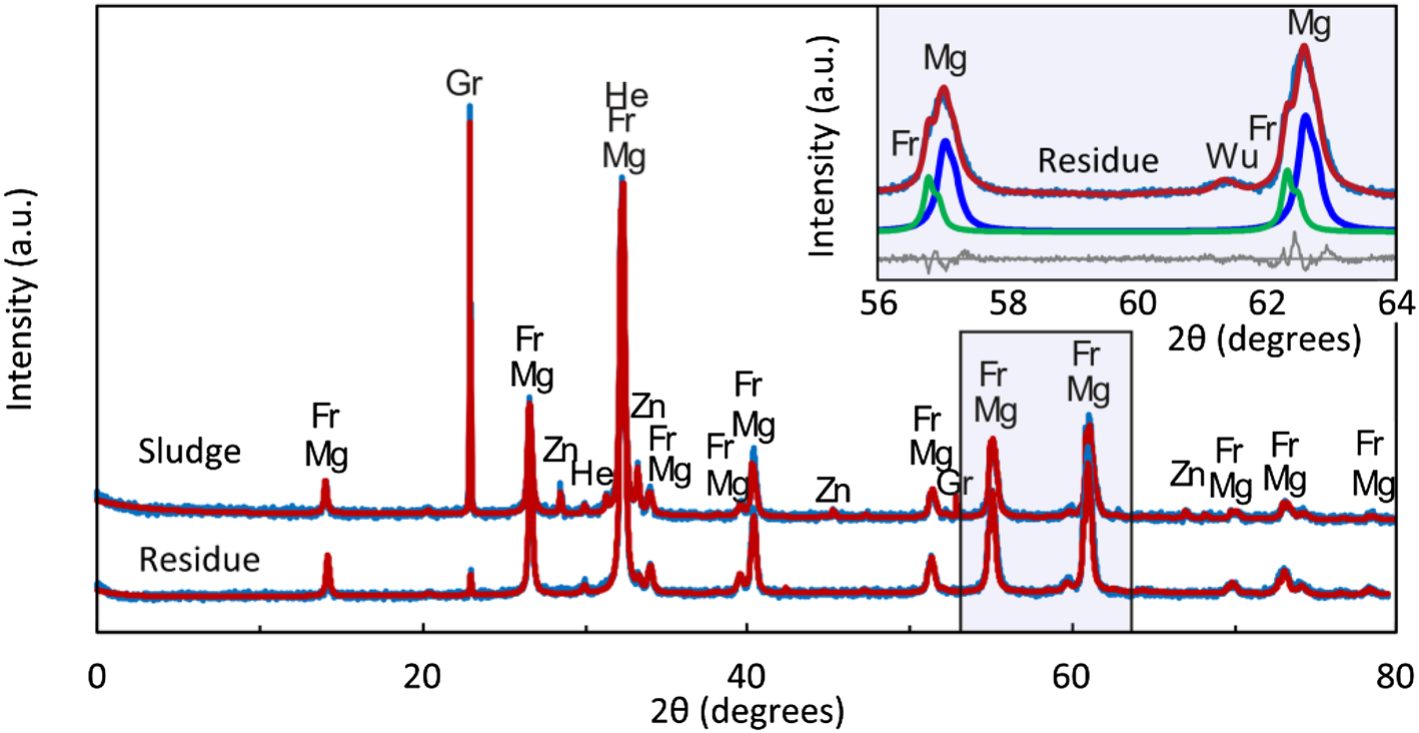
XRD patterns of the original sludge and the solid residue obtained by leaching in 0.01 M CH_3_COOH. Blue—magnetite, red—summary model, thin blue coinciding with red—measurement, grey—difference between the measurement and model. Green in the inlet—franklinite.

The comparison of the XRD patterns of the original sludge and the solid residue obtained by leaching in 0.01 M CH_3_COOH is shown in Fig. 4. It is clear that zincite (Zn) and part of wüstite (Wu) were leached, with zincite (Zn) not found in the residue. Franklinite (Fr), magnetite (Mg), and hematite (He) did not leach out and remained in the solid residue. The figure further shows that it is challenging to distinguish between magnetite (Mg) and franklinite (Fr), as their XRD records overlap significantly.

**Figure 5:**
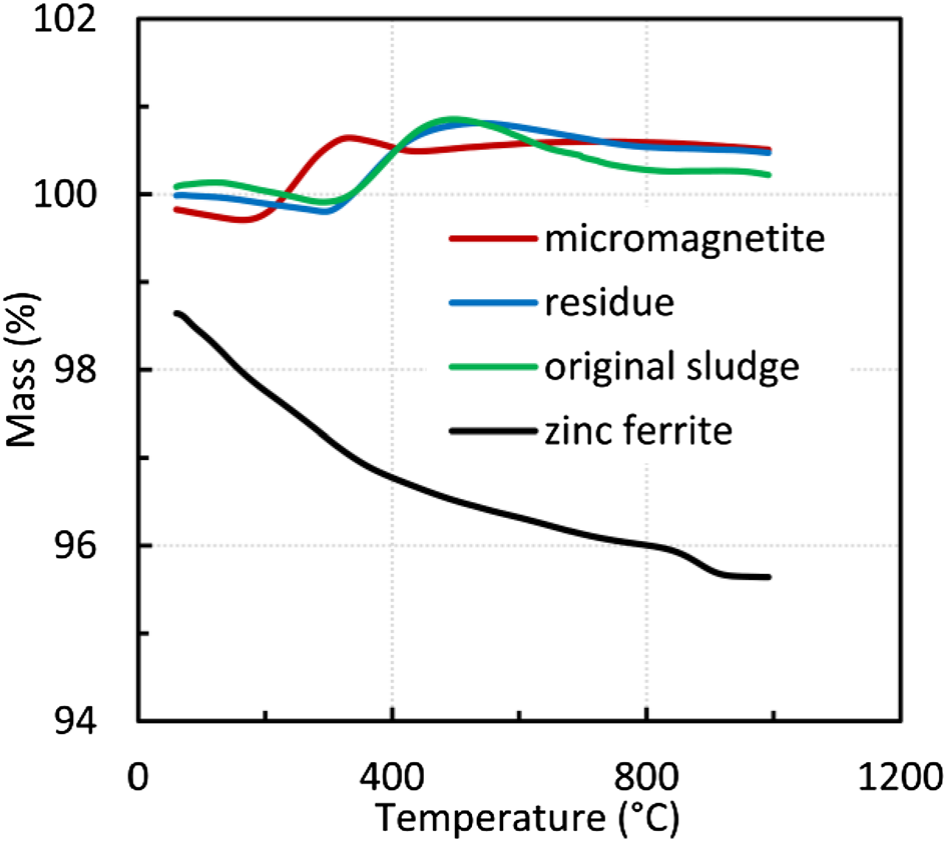
Thermal analysis of the sludge, zinc ferrite, solid residue, and micromagnetite, 10 °C min^−1^.

Thermal analysis was performed for the dried original sludge, the solid residue after le aching in a 0.01 M acetic acid solution, zinc ferrite, and micromagnetite (Fig. 5). Oxidation of Fe(II) to Fe(III) was evident in all samples except zinc ferrite. For micromagnetite, the reaction started at a lower temperature, probably due to the smaller particle size. For the original sludge sample and the solid residue after leaching, oxidation occurred in the temperature range of 310 to 530 °C. The resulting oxidation product was hematite, and the formation of maghemite cannot be excluded either. XRD analysis did not show its formation, but it may be caused by the small content of maghemite or its similarity to magnetite.

The original sludge and residue contained some minority components, e.g., graphite, whose oxidation started at 600 °C producing CO_2_ accompanied by mass loss. For zinc ferrite, the gradual weight loss in the initial stage can be attributed to some impurities or the release of gases and moisture trapped in the sample. Weight loss in the temperature range of 840–930 °C may be related to the gradual loss of zinc oxide, the decomposition of secondary phases, and the spinel phase formation^41^.

**Figure 6:**
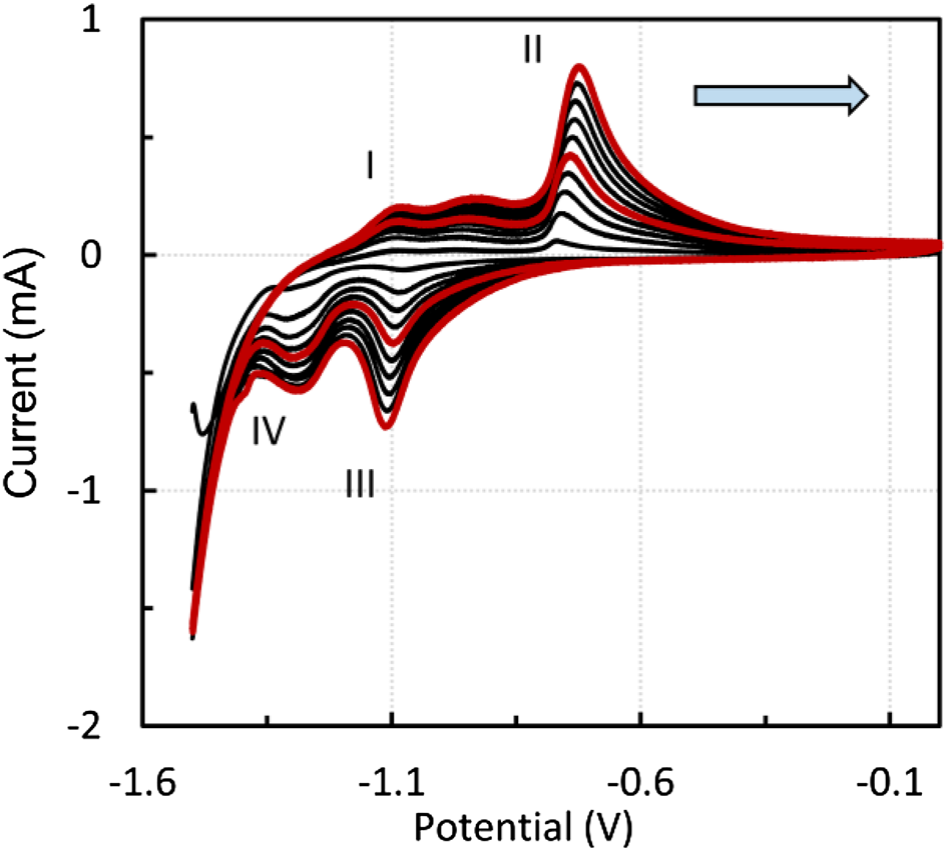
Voltammogram of CPE modified by the sludge residue after leaching in the 0.01 M acetic acid solution, 1 M NaOH, scan rate 10 mV s^−1^, positive direction, every fifth cycle is marked in red.

Figure 6 shows the voltammetric curves of CPE modified by the sludge residue after acetic acid leaching. Measurements were taken in 1 M NaOH solution at a scan rate of 10 mV s^−1^, starting from the anodic direction. The potential window was *E* = − 1.5–0 V, and a total of 10 cycles were performed. The electrochemical processes in the alkaline media were like those of the blast furnace sludge, as reported in^42^. The minor anodic peak I at about − 1.09 V can probably be attributed to reaction (1).

1 $$ {\text{Fe}}\left( {\text{s}} \right) + 2{\text{ OH}}^{ - } = {\text{Fe}}\left( {{\text{OH}}} \right)_{2} \left( {\text{s}} \right) + 2{\text{e}}^{ - } $$

A slight broad anodic peak at about − 0.93 V could be assigned to the oxidation of minority elements, i.e., manganese or lead. Further oxidation occurs approximately at − 0.7 V (peak II, reactions 2 and 3).

2 $$ 3{\text{Fe}}\left( {{\text{OH}}} \right)_{2} \left( {\text{s}} \right) + 2{\text{OH}}^{ - } = {\text{Fe}}_{3} {\text{O}}_{4} \left( {\text{s}} \right) + 4{\text{H}}_{2} {\text{O}} + 2{\text{e}}^{ - } $$

3 $$ 2{\text{Fe}}_{3} {\text{O}}_{4} \left( {\text{s}} \right) + 2{\text{OH}}^{ - } = 3{\text{Fe}}_{2} {\text{O}}_{3} \left( {\text{s}} \right) + {\text{H}}_{2} {\text{O}} + 2{\text{e}}^{ - } $$

**Figure 7:**
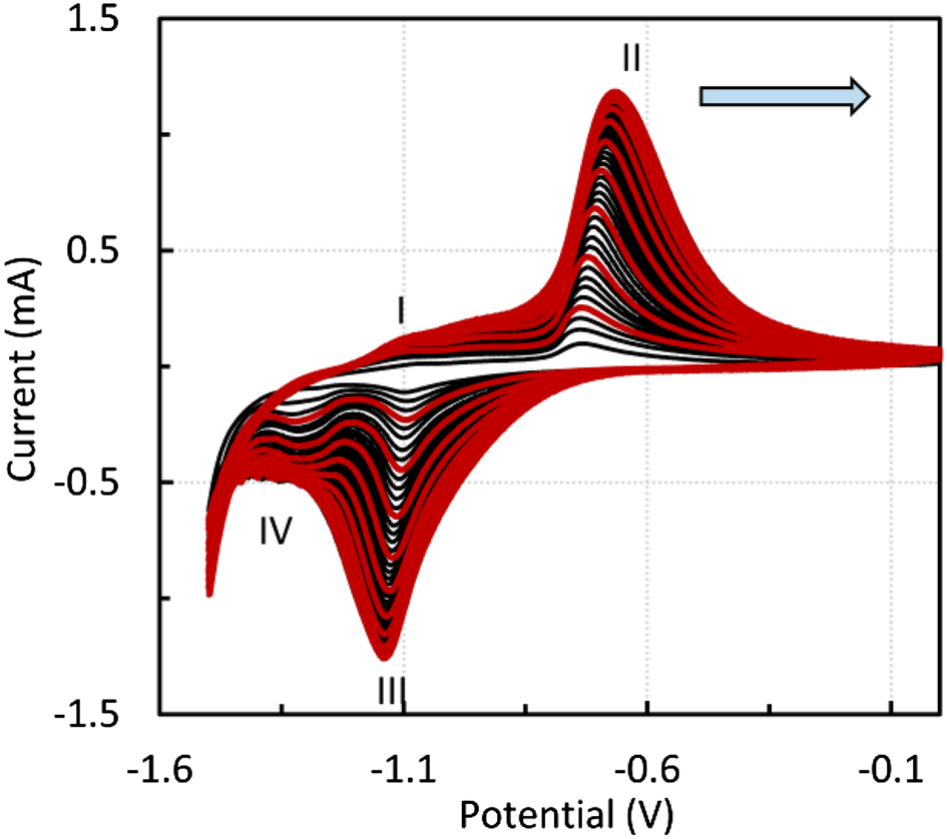
Voltammogram of CPE modified by the sludge residue after leaching in the 0.01 M acetic acid solution, 1 M NaOH, scan rate 25 mV s^−1^, every fifth cycle is marked in red.

At lower scan rates, two reduction peaks were distinguished. At − 1.1 V, the reduction of Fe(III) to Fe(II) occurs (reverse reactions 2 and 3, peak III). The cathodic peak IV at about 1.24 V probably corresponds to reverse reaction 1. When using higher scan rates, only one anodic and one cathodic peak can be observed (Fig. 7, 50 cycles, 25 mV s^−1^), and the redox reactions of the individual oxidation states cannot be detected.

**Figure 8:**
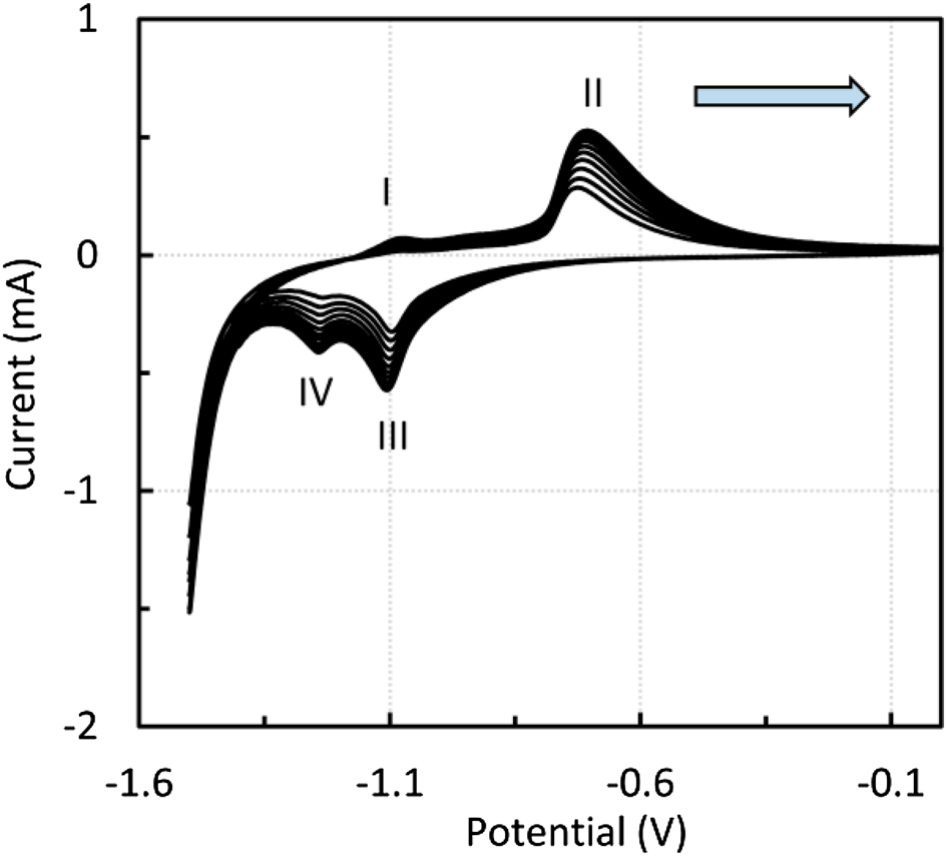
Voltammogram of CPE modified by micromagnetite, 1 M NaOH, scan rate 10 mV s^−1^.

**Figure 9:**
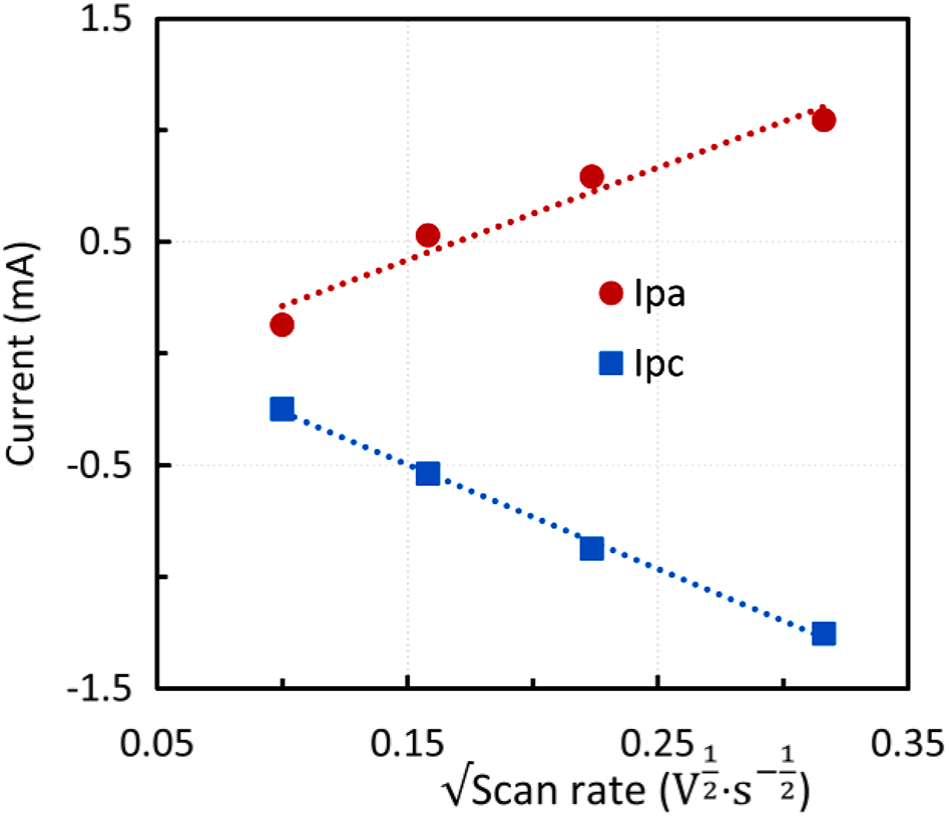
Effect of the scan rate on the anodic and cathodic peak height—the second cycle, micromagnetite-modified CPE.

Because the residue mainly contains magnetite and zinc ferrite, similar experiments were performed with CPE modified by micromagnetite and zinc ferrite. Figure 8 shows the voltammetric curves of CPE modified by micromagnetite. The voltametric cycling was carried out in the potential window of *E* = − 1.5–0 V at a scan rate of 10 mV s^−1^, starting with the anodic sweep. In total, 10 cycles were performed in 1 M NaOH solution. Figure 9 shows that micromagnetite-modified CPE behaves similarly to a reversible inert electrode immersed in analyte solution because the dependence of the anodic and cathodic peak heights on the square root of the scan rate is nearly linear and obeys the Randles–Ševčík equation.

**Figure 10:**
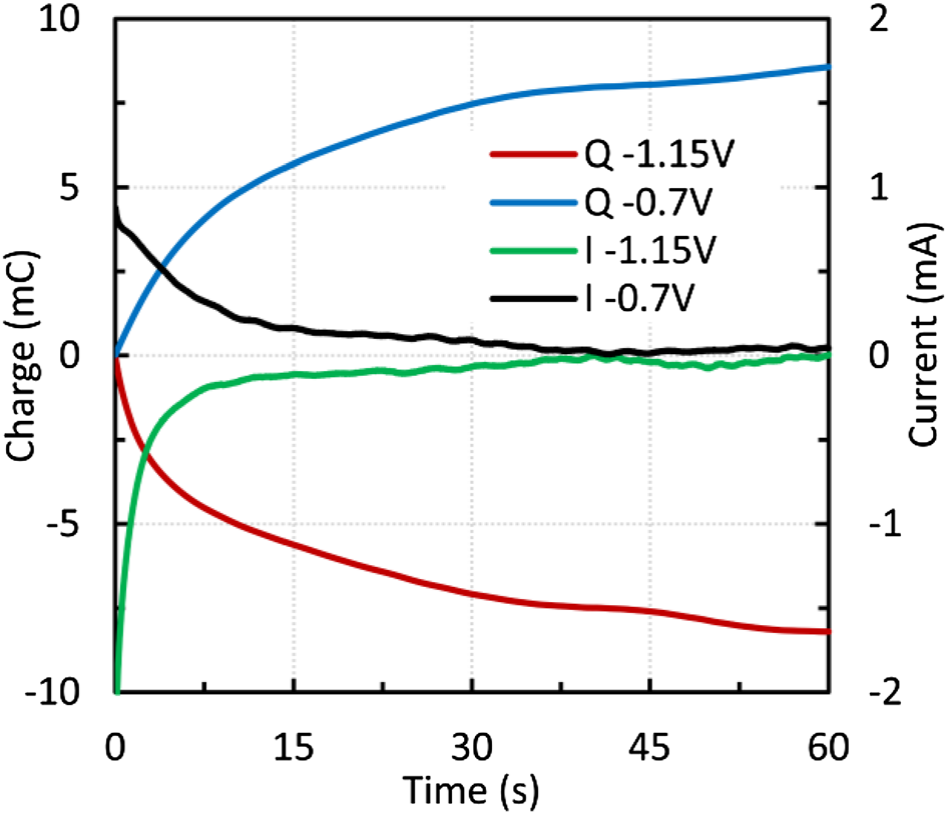
Micromagnetite—chronocoulometric curves after 7 voltammetric cycles at 10 mV s^−1^.

**Figure 11:**
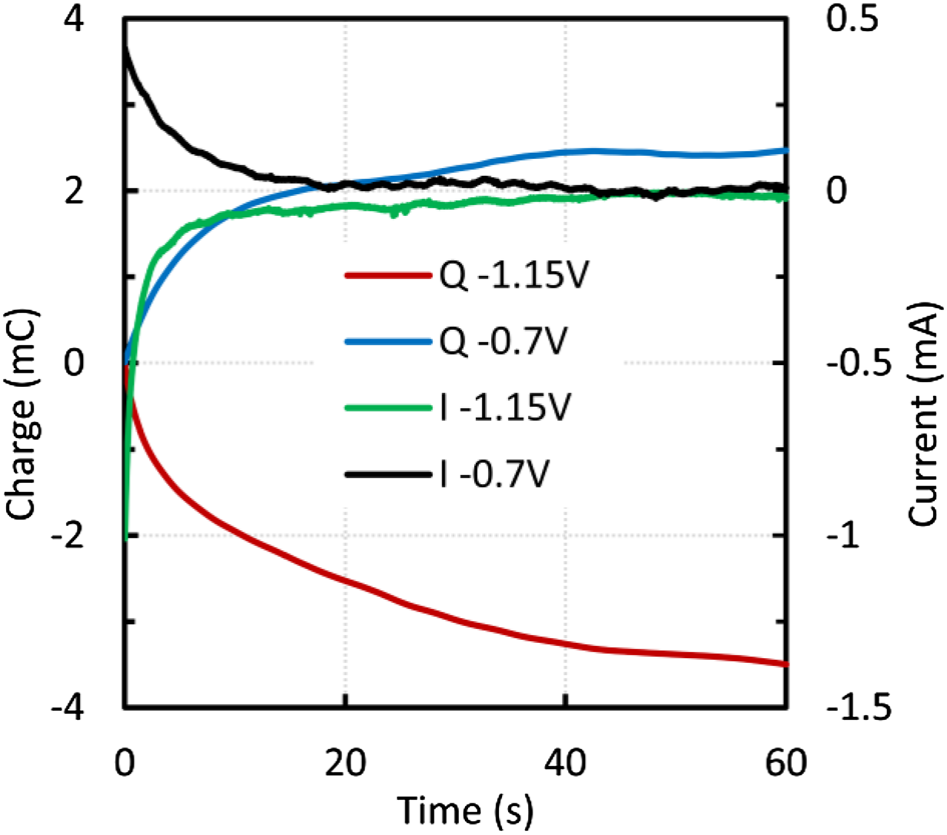
Micromagnetite—chronocoulometric curves after 10 voltammetric cycles at 50 mV s^−1^.

Chronocoulometry was used for further investigation. Chronoculometric curves were measured after each voltammetric cycle at 10 mV s^−1^. After approximately 7 cycles, the peak heights did not increase significantly. As can be seen in Fig. 10, the current values after 60 s were close to zero, indicating that most of the available surface was oxidized or reduced. The charge values after oxidation and reduction were almost the same, providing solid evidence of a reversible reaction. On the other hand, in the case of a scan rate of 50 mV s^−1^, the peak values kept increasing, and the charge values were different (Fig. 11).

**Figure 12:**
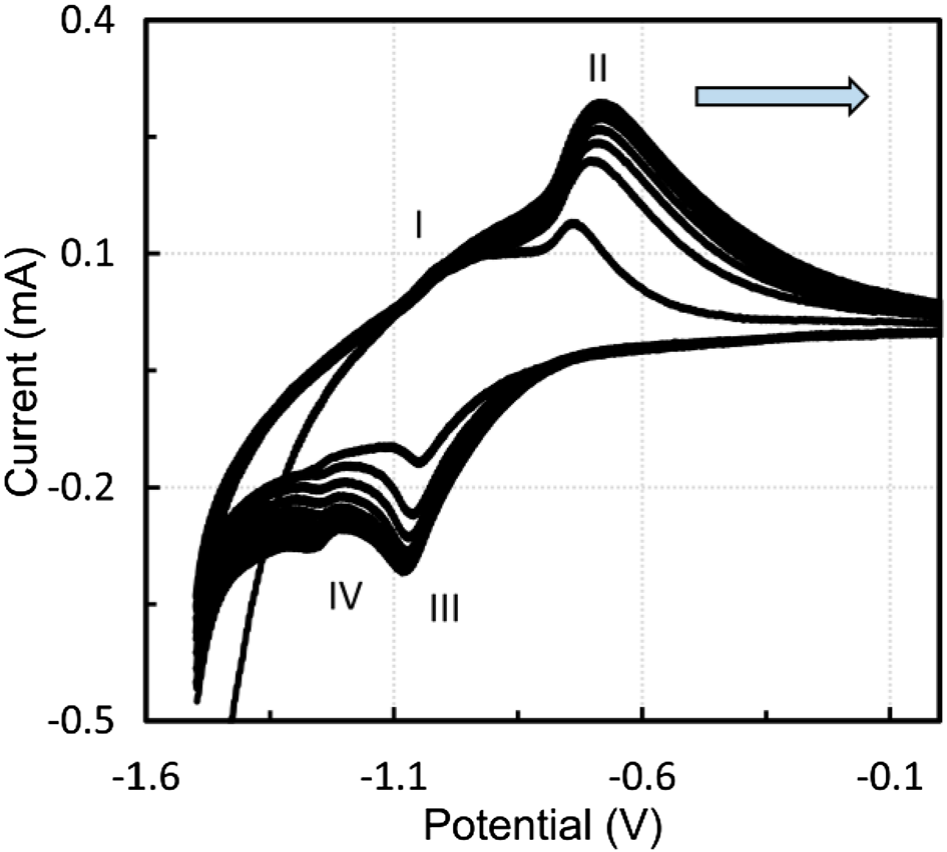
Voltammogram of CPE modified by zinc ferrite, 1 M NaOH, scan rate 10 mV s^−1^.

Figure 12 shows the voltammetric curves of zinc ferrite at a scan rate of 10 mV s^−1^ in a potential window of *E* = − 1.5–0 V, starting with the anodic direction. The peaks are in positions similar to those of magnetite, and the first anodic peak is not as significant. It is clear from Fig. 12 that the redox processes during the voltammetric cycling concern only iron, with the zinc redox processes lying outside the monitored potential window.

**Figure 13:**
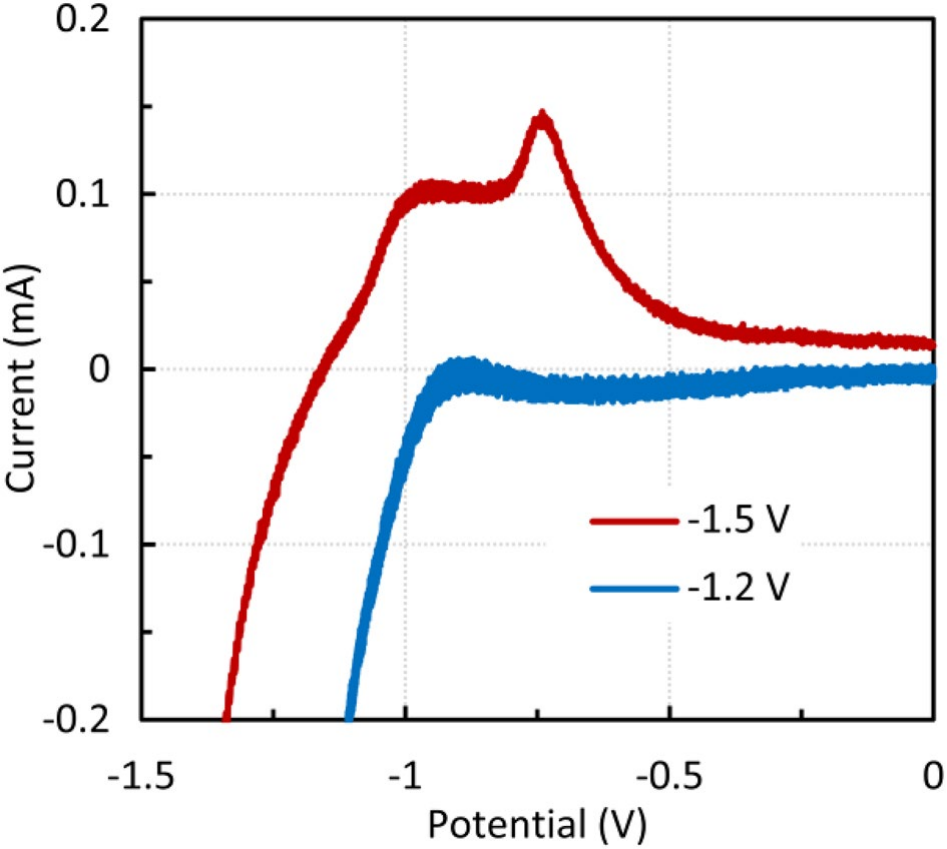
Effect of the starting potential on the anodic peak height, zinc ferrite, 1 M NaOH.

It is worth noting that if anodic sweeps start at higher potentials, e.g., − 1.2 V, anodic peaks are not formed since the reduction reaction predominates, as shown in Fig. 13. Once Fe(0) is formed due to hydrogen evolution at a potential of approximately − 1.5 V, the anodic peak can be observed. The presence of metallic iron was proved in^43^. When starting in the cathodic direction from 0 to − 1.5 V, a sufficient amount of Fe(II) is formed and anodic peaks also occur. A similar result was found for magnetite. The dependence of the anodic and cathodic peak heights on the square root of the scan rate was also nearly linear, as was the case for micromagnetite.

**Figure 14:**
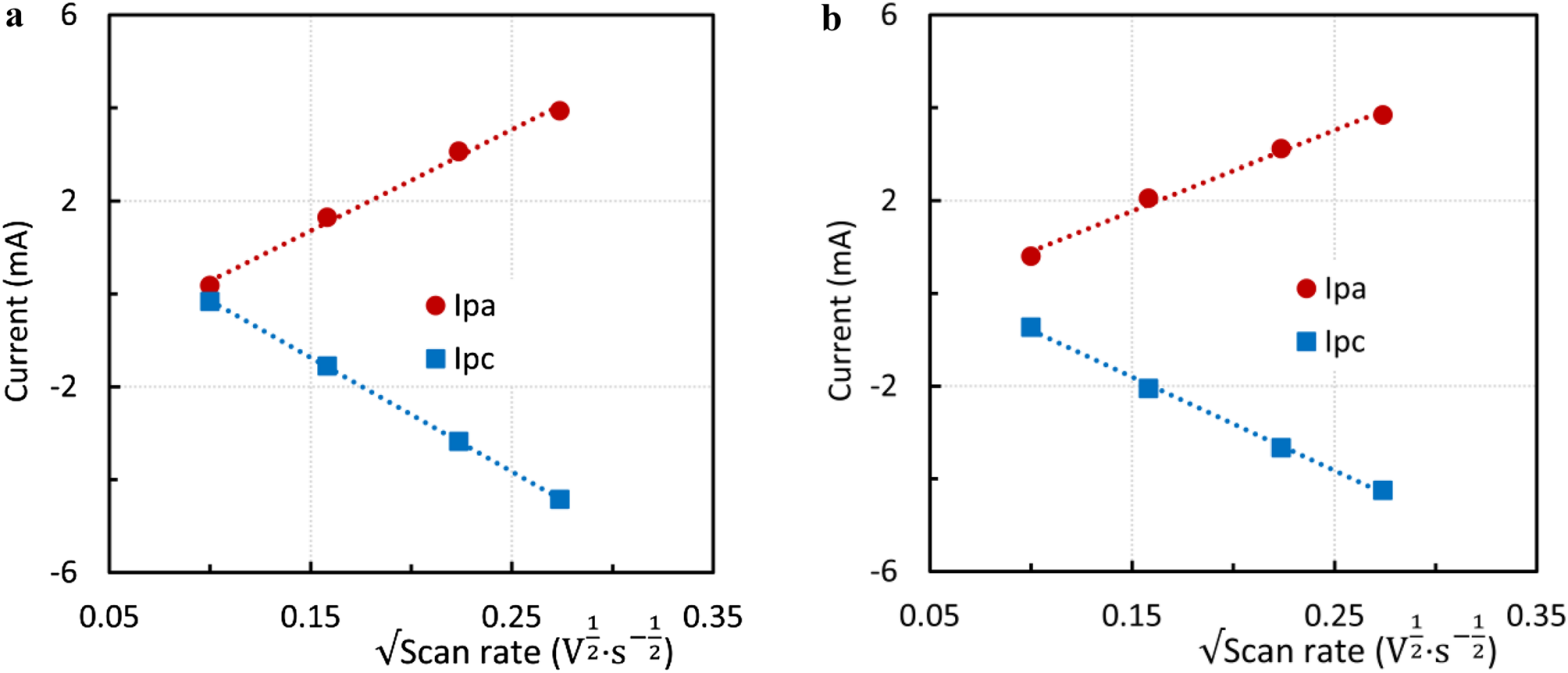
(**a**) Effect of the scan rate on the anodic and cathodic peak height—second cycle, sludge residue (**b**) Effect of the scan rate on the anodic and cathodic peak height, the tenth cycle, sludge residue.

**Figure 15:**
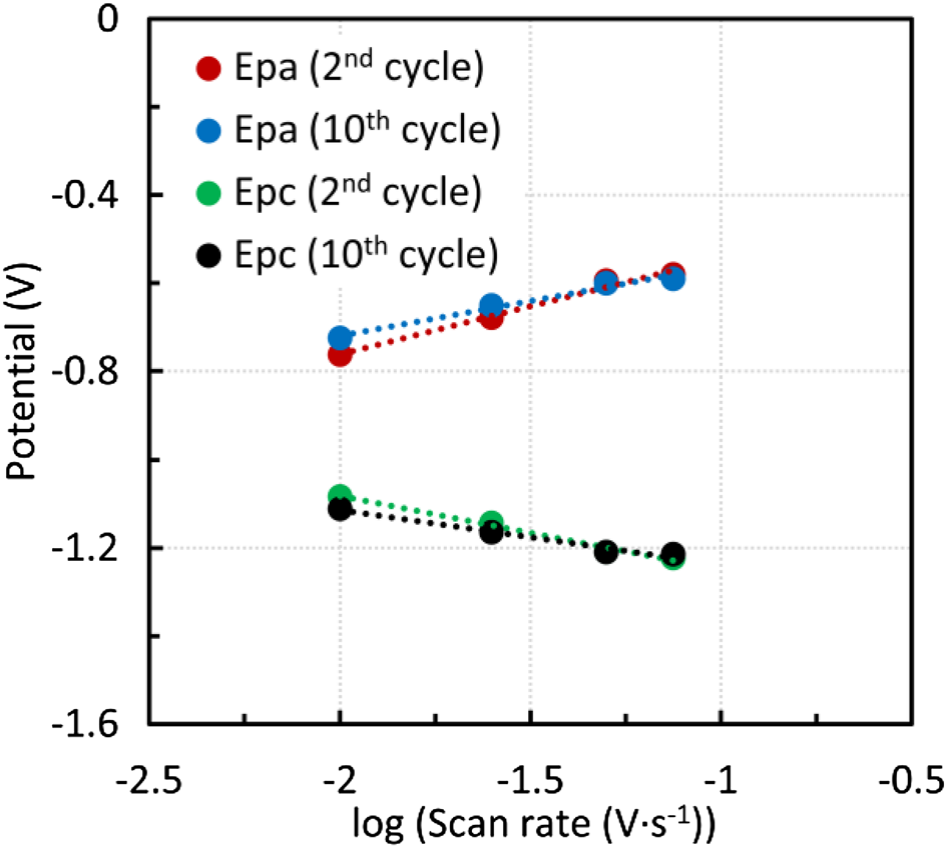
Effect of the scan rate on the position of the peak potentials, sludge residue.

The findings were applied to CPE modified by steelmaking sludge after acid leaching. The ratio of the anodic and cathodic peak heights was close to one. In this regard, the process can be characterized as quasireversible. The dependences of the anodic and cathodic peak heights on the square root of the scan rate were almost linear (Fig. 14a,b). However, the absolute values of the angular coefficient for the anodic and cathodic parts were not identical*.* Anodic and cathodic peak heights gradually increased with increasing number of cycles. This may have been caused by an increase in the electrode surface as a result of solution penetration and elution of the inert material. The effect of the scan rate on the peak potential can be seen in Fig. 15. The anodic peak potential was shifted positively, whereas the cathodic peak potential was shifted negatively, indicating a quasi-reversible process. For a surface-controlled redox process where the distance between the peaks is greater than 0.2 V n^−1^, the relationship between the peak potential $E_{p}$ and the scan rate can be expressed by Eq. 4^44^.

4 $$ E_{p} = f\left( {\log v} \right) $$

The dependences were almost linear. Nevertheless, an evaluation of the electron transfer coefficient or the rate constant would be pointless as this was not a process in the analyte solution. In addition, the carbon paste electrode modified by the electroactive compounds behaved similarly.

## Conclusion

Acetic acid was used for leaching the steel-making sludge. The zinc contained in the zincite was extracted almost selectively using dilute acetic acid, and the selectivity decreased with increasing acid concentration, liquid/solid ratio, and time. It turned out that the best results were obtained for 0.01 M acetic acid, a solid-to-liquid ratio of 500 and a time of 1–3 h. Mineralogical analysis showed that zincite was removed, opening the way for the use of leach liquor to prepare photocatalysts. Thermal analysis, Raman spectroscopy, cyclic voltammetry, and chronocoulometry were used to characterize the solid residue after acid leaching, and the results were compared with the original sludge sample and the pure components, micromagnetite and zinc ferrite. In addition, thermal analysis showed a high thermal stability of the leached material compared to that of pure zinc ferrite. For all samples except zinc ferrite, oxidation to hematite and maghemite was confirmed by thermal and XRD analysis. The oxidation of micromagnetite was shifted to a lower temperature as micromagnetite had the smallest particle size. Raman spectroscopy confirmed the presence of magnetite and franklinite as the main crystalline phases of steel-making sludge.

The results of the electrochemical experiments were similar for all materials studied. The dependence of the anodic and cathodic current maximum on the square root of the scan rate was linear when using the modified carbon paste electrode, as was the case for the reversible process with an inert electrode in the analyte solution. Further, for the modified carbon paste electrodes, the current increased with increasing number of cycles as the electrode surface area enlarged. The ratio of anodic to cathodic peak was close to one, indicating a quasi-reversible process. On the other hand, the peak position was rather dependent on the scan rate. Only low scan rates were suitable for the investigated reactions, because from 25 mV s^−1^ the individual peaks merged. After a few cycles depending on the scan rate in the potential window of − 1.5–0 V versus SCE, the system reached a quasi-equilibrium.

The fundamental electrochemical characteristics of the solid residue obtained after acid leaching can provide a basis for further use of this material, for example, as a contrast agent for magnetic resonance imaging thermometry, in magnetic fluid hyperthermia therapy, and in microwave absorbers, gas sensors and semiconductor photocatalysts.

It was verified that thermal analysis, cyclic voltammetry, and chronocoulometry could be complementary methods to XRD analysis for the same particle size. The solid residue consists mainly of zinc ferrite and magnetite. This material will be studied concerning the preparation of hybrid nanocomposites with unique magnetic properties and catalysts. The investigation of the electrochemical behaviour of iron compounds will continue, given the growing importance of environmentally friendly technologies such as green metallurgy.

## Data Availability

The datasets generated and/or analysed during the current study are not publicly available because they are a part of the project but are available from the corresponding author on reasonable request.
